# Patterned Microstructure Fabrication: Polyelectrolyte Complexes vs Polyelectrolyte Multilayers

**DOI:** 10.1038/srep37000

**Published:** 2016-11-10

**Authors:** Meiyu Gai, Johannes Frueh, Valeriya L. Kudryavtseva, Rui Mao, Maxim V. Kiryukhin, Gleb B. Sukhorukov

**Affiliations:** 1 Micro/Nano Technology Research Centre, Harbin Institute of Technology, Yikuang Street 2, Harbin 150080, China; 2 School of Engineering and Materials Science, Queen Mary University of London, Mile End, Eng, 215, London, E1 4NS, United Kingdom; 3 National Research Tomsk Polytechnic University, RASA Center in Tomsk, Department of Experimental Physics, Tomsk, 634050, Russia; 4 Institute of Materials Research and Engineering (IMRE), A*STAR, 2 Fusionopolis Way, Innovis, 08-03, 138634, Singapore

## Abstract

Polyelectrolyte complexes (PEC) are formed by mixing the solutions of oppositely charged polyelectrolytes, which were hitherto deemed “impossible” to process, since they are infusible and brittle when dry. Here, we describe the process of fabricating free-standing micro-patterned PEC films containing array of hollow or filled microchambers by one-step casting with small applied pressure and a PDMS mould. These structures are compared with polyelectrolyte multilayers (PEM) thin films having array of hollow microchambers produced from a layer-by-layer self-assembly of the same polyelectrolytes on the same PDMS moulds. PEM microchambers “cap” and “wall” thickness depend on the number of PEM bilayers, while the “cap” and “wall” of the PEC microchambers can be tuned by varying the applied pressure and the type of patterned mould. The proposed PEC production process omits layering approaches currently employed for PEMs, reducing the production time from ~2 days down to 2 hours. The error-free structured PEC area was found to be significantly larger compared to the currently-employed microcontact printing for PEMs. The sensitivity of PEC chambers towards aqueous environments was found to be higher compared to those composed of PEM.

3D Polyelectrolyte complexes (PECs) have been investigated since the 1920 s when coacervation phenomena were first described^1^,^2^. The investigation was continued for decades thereafter where PECs were found not to be very useful for fabrication of large products (except for the food industry) due to processing difficulties and their sensitivity to environmental influences like salt, water or humidity^3^. In the 60 s, the possibility of PEC casting as well as possible applications of the obtained thin films were discussed by Michaels^4^. Recently the Schlenoff group introduced a novel way for PEC extrusion based on Saloplastics and even a PEC swelling-based microparticle production method^5^,^6^,^7^. However, micro or nanostructuring of flat PEC films was never reported.

In the last twenty-five years, 2D layered PECs also known as Polyelectrolyte multilayer films (PEMs) are investigated and are now frequently employed as a functional surface coating due to their stimuli responsiveness, electrical as well as mechanical properties^8^. Such coatings are now commonly produced utilizing Layer-by-Layer (LbL) self-assembly methods *via* either the time consuming dip-coating^9^,^10^ or less time but more solvent consuming spraying approach^11^. Due to their stimuli responsiveness and simplicity of productions, such PEM coatings are used in a variety of applications, especially in the field of surface mediated drug delivery, antibiofouling or implants^12^,^13^. PEM films with defined surface micro- or nano-structuring (being called either 3D or 2.5D^14^,^15^,^16^,^17^) have been demonstrated and used as sensors, in optics, electronics, bio-electronics^18^,^19^,^20^,^21^. Selective PE adsorption effects were used to create a 2D PEM pattern^22^. Structuring homogeneous PEM films, in a subsequent step, the pattern is usually created by microcontact printing, or by destroying or lifting off undesired regions^17^,^23^,^24^,^25^. For chamber-like PEM patterns, only approaches in which one performs PEM assembly on a patterned polymeric template which later is dissolved (sacrificed) as well as microcontact printing were successfully employed until now^26^.

In this publication we present a method for structuring a macroscopic PEC aggregate into a macroscopically-structured 2D but microscopically-patterned 3D forms. This patterning approach exploits thermo-, hydro- and saloplastic properties of PECs and allows heat-induced re-arrangement and shaping of the PECs structure prior to drying. We present additionally a comparative analysis of our approach with currently established PEM films microstructuring^15^,^16^,^17^. The approaches are compared in terms of production time, efficiency, printing pressure, elastic modulus of the resulting PEC and final pattern structure. Effects that are known to have an influence on resulting PEC structure like: ionic strength of the PEC production solution; embedding of particles, age of PEC stored in solution prior to processing, molecular weight of the PE and pH of the production solution will be addressed in subsequent publications. Please note that throughout this publication the word “mould” is used for micro-structured Poly(dimethylsiloxane) (PDMS, silicone rubber) since the PEC structuring is a casting process with small applied pressure. This nomenclature is kept despite the PEM microcontact printing using the same PDMS patterns as “stamps”.

## Results

### Micro-structured PDMS mould

PDMS moulds for microcontact printing were produced by casting a curable PDMS precursor onto a silicon master, curing PDMS and lifting it off, similar to ref. 27 (see also Methods and materials as well as and SI Fig. S1. for details). This PDMS mould (1) is the negative replica of the silicon master which contains micro-wells of round, square or truncated pyramidal shape. On the contrary, the PDMS mould (2) made from PDMS mould (1) has same micropillar patterns like silicon master.

### Polyelectrolyte Multilayers (PEM) Microstructures Made by Layer-by-layer (LbL) Self-Assembly and Microcontact Printing Approach

Using PDMS mould 1 (Supplementary Information Fig. S1(c–e)), one can create PEM microchambers (Supplementary Information Fig. S1(b,f–h)). This is done by layer-by-layer assembling (for assembly conditions, see Methods and Materials) of PEM on PDMS mould 1 which has micro-well like patterns on the surface, followed by transferring the patterned 3D hollow PEM structures to a substrate (e.g. glass slide or silicon wafer). Due to different adhesive forces between PEM-PDMS and PEM-silica (or glass slide) substrate, the PEM microchambers are transferred to the substrate with higher surface energy (for printing results see Supplementary Information Fig. S1 and refs 10,17 and 28).

### Free- standing Polyelectrolyte Complexes (PEC) Microstructures Made by One-Step Casting with Small Applied Pressure

#### PEC Precipitation Behavior and Polyelectrolyte Ratios

Mixing oppositely charged polyelectrolyte (PE) solutions (for concentrations and details see Methods and Materials) causes the formation of polyelectrolyte complexes (PEC), which instantly turn the solution turbid. The precipitation properties of the PEC varied vastly between the PE types. Strongly charged PEs like poly(styrenesulfonate) (PSS) and poly(diallyldimethyl ammonium) (PDDA) precipitated and agglomerated readily and were easy to collect in the used conditions, since they formed a single large PEC aggregate within ~10 min of stirring time (500 RPM). The PSS-PDDA PEC displayed in FTIR-spectra peaks which correspond to both types of PE, polyanion and polycation, as shown in Supplementary Information Fig. S2(a). It is noted that for this measurement most PDDA peaks are not strongly pronounced compared to PSS, which offers only 1 peak with medium strength (see Supplementary Information Fig. S2).

Since the exact molar absorption coefficients of the functional groups are not known, and most literature states only “medium” or “strong” absorption we decided to quantify the molar ratios of PEC via volumetric titration of corresponding supernatant solutions^29^,^30^. As can be seen from Supplementary Information Fig. S2(c), an excess of polycations of 23 ± 3%, was determined, which is in agreement with the added excess of polycations of 22%. This means that the PSS-PDDA PEC has a stoichiometric concentration of PSS and PDDA, which is in contrast with recent reports on excess polycations in PEM formed at high ionic strength media (>0.25 M) in PEM, and is most likely due to different density and complex formation processes of PEC and PEM^5^,^31^,^32^,^33^,^34^.

PECs formed by weak polyacids/polybases on the contrary showed a strong pH dependent precipitation behavior and undergo decomplexation at low (<5) or high (>8–9) pH values, depending on the charge of used groups. The reason for the PEC decomplexation is decrease of charge density on either polycation or polyanion due to suppressed dissociation of the acidic or basic groups. This observation is in accord with literature^35^. Precipitation behavior of poly(allylamine hydrochloride) (PAH)–poly(acrylic acid) (PAA) complexes was similar to PSS-PDDA described only at pH 5.5–6.5, whereby the precipitate also agglomerated and formed one easy-to-collect big PEC aggregate within ~10–20 mins of stirring at 500 rpm. Therefore only these conditions were used to produce PAA-PAH PEC for casting with small applied pressure.

Upon volumetric titration an excess of PAH of up to 28% is determined, as shown in Supplementary Information Fig. S2(d). Finding an excess of PAH is surprising, especially since PAA was added in monomer excess of 23%. Using fitted FTIR peak area ratios of (peaks of medium strength) for polyanion (PAA) to polycation (PAH) a ratio of 1.19–2.28:1 (depending on used peak in Supplementary Information Fig. S2(b)) was determined in the PEC. It is noted that for all determinable peak ratios an excess of PAA was determined. The excess of PAA in PEC is based on volumetric titration 1.85 ± 0.18:1 which is in good agreement with the averaged excess of PAA for all fitted FTIR peaks which is 1.74 ± 0.25:1. The detected excess of PAA (mentioned in main article) within PEC is most probably due to the slightly acidic pH upon complexation which caused a significant portion of PAA acidic groups to be protonated. Therefore the PAA, despite being added in excess is mostly neutralized and therefore does not contribute to the complexation of PAH. IR spectra of PSS-PDDA as well as PAA-PAH PECs can be observed in Supplementary Information Fig. S2(a,b) while volumetric results are presented in SI Fig. 2(c,d).

Contrary to previous reports^36^,^37^, the poly(ethylenimine) (PEI)–PAA complex was barely precipitate-able at all tested pH (pH 2–9) and collected either from the foam or from the magnetic stirrer. The subsequent PEC was extremely sticky and hard to process or transfer. Similar sticking effects were determined for PSS-PEI polyelectrolyte complexes. Since pH significantly affects the internal charge as well as PE ratio of weak polyacid and polybases in PEC^35^,^38^ we attribute these pH independent sticking and bad processing effects to the chain length of PEI. The chain length as well as the branching degree of PEI is significantly higher than the one of PDDA or PAH. The water content of all weak as well as strong PE containing wet PEC aggregates was 60 ± 1.5%.

#### Properties of PEC after Casting with Applied Pressure

**Figure 1:**
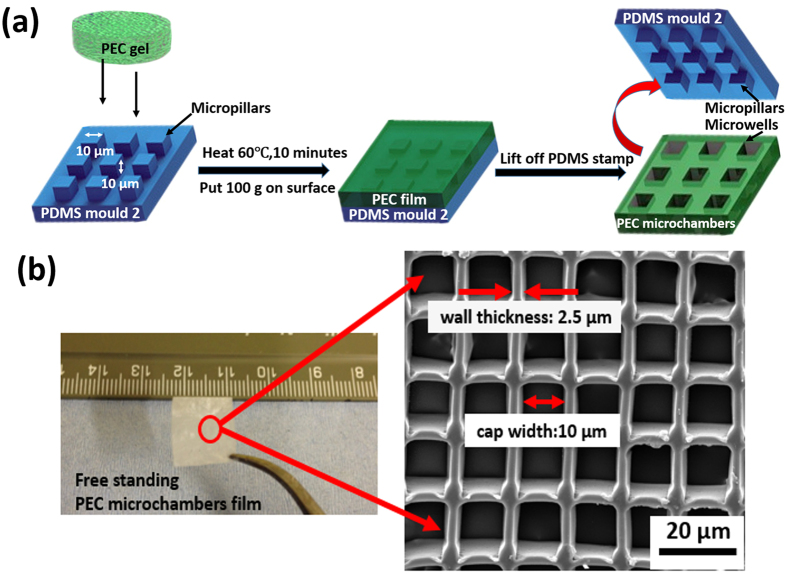
(**a**) Scheme illustrating the fabrication of polyelectrolyte complexes (PEC) micro chambers by pressing the PEC against patterned PDMS mould 2 which has micropillars at elevated temperatures followed by subsequent drying and mould lift-off; (**b**) Image of free-standing PEC (PSS-PDDA) patterned film and SEM image of the film with array of square microchambers.

The produced PECs, no matter of which type, can readily be cast and pressed into the desired structures by pressing it with a flat polymer (PDMS) foil onto either PDMS mould 1 (with microwells) or PDMS mould 2 (with micropillars), heating (while it is still wet) to 60–70 °C, removing the foil and subsequent drying the PEC at elevated temperature, to lift off the mould, as shown in Fig. 1.

**Figure 2:**
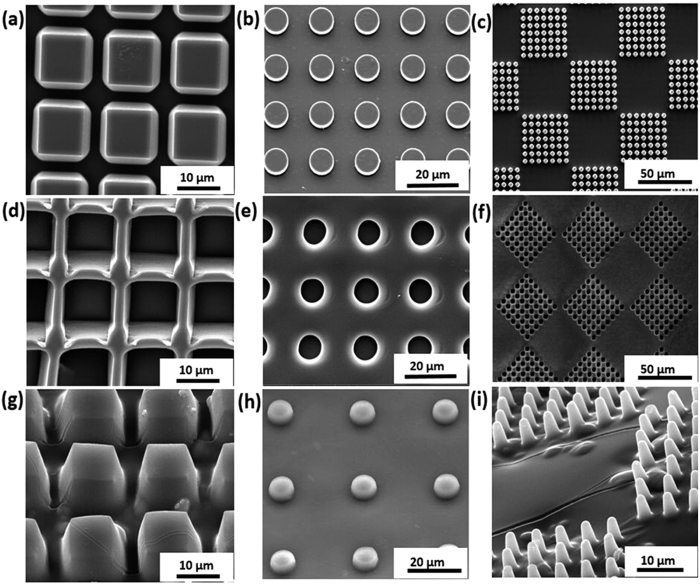
(**a**–**c**) SEM images of PDMS moulds 2 with micropillars made by differently patterned (square, round and truncated pyramidal shape) PDMS moulds 1; (**d**–**f**) SEM images of PEC (PSS-PDDA) films with imprinted hollow microwells produced by differently patterned PDMS moulds 2; (**g**–**i**) SEM images of PEC (PSS-PDDA) films with imprinted filled micropillars produced by differently patterned PDMS moulds 1.

The resulting PECs are stable-freestanding films. All produced PECs displayed the same quality of casting. An example of resulting microstructures for PSS-PDDA PEC is shown in Fig. 2. As can be clearly seen all structures are negative replicas of the casting mould, whereby the mould shape can be arbitrary. The smallest feature size used in the work was 2.5 μm (the width of the walls), with a height-to-width aspect ratio of 4 which is shown in Fig. 2.(d). Observed deviations of cast structures from the mould were <1 μm and could be attributed to deformations in PDMS mould under applied pressure. Utilizing printing pressures far below the elastic deformation limit of PDMS (pressure ~single to dozens of Pa, instead of kPa), an embossing replication limit similar to surface-tension based deformations of PDMS (~100–200 nm) was achieved in this study, which is in line with previous PDMS printing records^27^,^39^. Pressure was however necessary to create microstructured PEC films with a thin “cap” on the top (also known as residual layer in classical imprinting); the average replica deviation was therefore ~200–500 nm.

### Comparison of PEC and PEM Microchambers

#### Produced Micro-patterns and Production Time

**Figure 3:**
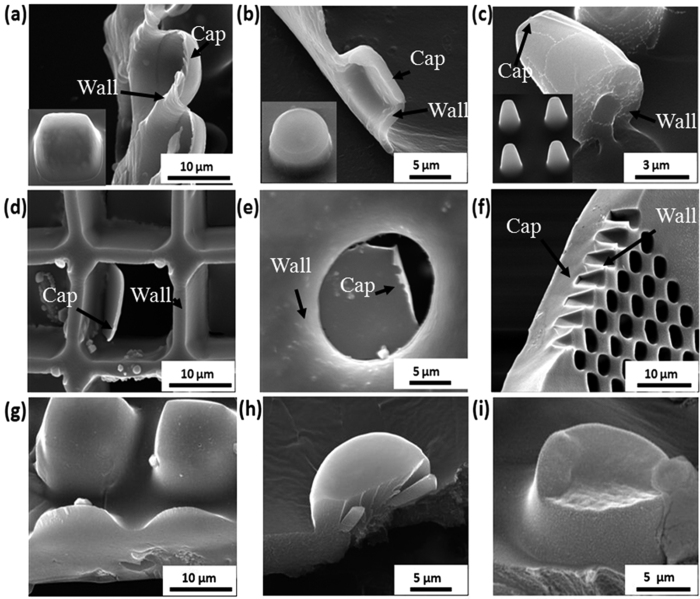
“Wall” and “cap” thicknesses of PEM and PEC microchambers. (**a–c**) SEM images of printed hollow PEM (PSS/PDDA)_60_ microchambers of different structures on a silicon wafer, the “wall” and “cap” thickness: (**a**) square (~500 nm), (**b**) round (~200–300 nm) and (**c**) truncated pyramid shaped micropillars (~1 μm) (similar cap and wall thickness); (**d**–**f**) SEM images of hollow PEC (PSS/PDDA) microchambers with (**d**) square (~600 nm), “wall” thickness (~2 μm), (**e**) round pattern “cap” thickness (~500 nm), (**f**) microchambers arranged in truncated pyramidal shape with ~9 μm (measurement angle being ~45°, therefore measurement on image needs to be corrected for measurement angle) cap width. (**g**–**i**) SEM images of PEC filled micropillars made from PDMS mould 1, (**g**) square micropillars, (**h**) round micropillars, (**i**) truncated cones.

As Supplementary Information Fig. S1(f–h) and Fig. 3(a–c) show, both (PSS-PAH)_60_ and (PSS/PDDA)_60_ (60 bilayers of PE) films produced from PDMS mould 1 consist of isolated hollow microchambers sealed onto a substrate. If one uses PDMS mould 1 for PEC production, PEC patterns of the same shape like the PEM microchambers will be produced, but these patterns are filled pillars instead of hollow chambers as shown in Figs 2(g–i) and 3(g–i). Figure 2(d–f) is showing PEC films made from PDMS mould 2 exhibiting microwells of the negative structure compared to pillars produced by mould 1. These films are free standing films due to the PEC backside being their support (Fig. 3(d–f)).

The production time required for PEM assembly of 60 bilayers was 32 hours, via automated dipping approach with additional 1 hour printing (others reported printing times up to 12 hours, for larger homogenous areas)^10^. PEM production time is much longer compared to the 1–2 hours required for the PEC printing approach. In addition, the resulting PEM microchambers were free of defects on areas less than 0.5 cm^2^, probably due to uneven pressure distribution during printing, compared to a perfect PEC printing on 10.2 cm^2^ (the limit of our moulds, as shown in Supplementary Information Fig. S3.). Coating the PEM films on patterned PDMS mould 2 resulted in non-transferrable PEM structures, due to a too low contact area between glass-PEM compared to PDMS-PEM.

#### PEC and PEM Microchambers “Cap” and “Wall” Thickness

The PEC microchambers backside “cap” thickness can be readily adjusted by varying the pressure applied to the PDMS on top of a PEC aggregate as shown in Supplementary Information Figs S3 and 4. The “wall” thickness of PEC microchambers rely on the PDMS mould patterns itself. On the contrary, the PEM microchambers “cap” and “wall” thickness are both dependent on the numbers of the deposited PEM bilayers.

The PDMS mould sides are compressed linearly, as PDMS being a linear elastic material in the utilized pressure range^40^,^41^. The backside thickness was found to be adjustable from an arbitrary thick PEC sheet behind embossed structures all the way down to zero (free grating with no caps on the cast PEC structure, see Supplementary Information Fig. S4.). PEM (PSS-PDDA)_60_ microchambers’ caps with thickness 500–800 nm can be seen in Fig. 3(a,b). PEC microchambers of similar caps’ thickness (~600 nm) are shown on Fig. 3(d,e). Thicker PEC caps of ~9 μm mentioned can be seen in Fig. 3f.

**Figure 4:**
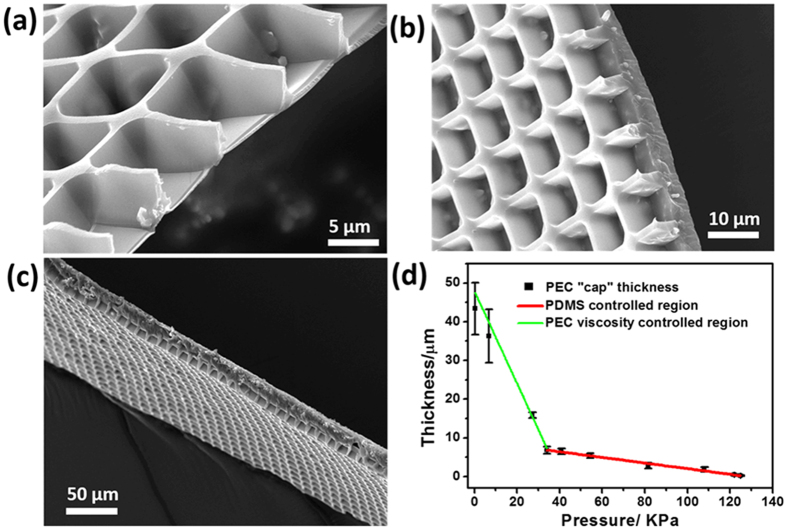
Influence of pressure on PEC microchamber “cap” thickness in dry condition, (**a**–**c**) SEM images show PECs cap thickness produced at 110 kPa, 55 kPa and 30 kPa pressure, (**d**) the figure displays the pressure to thickness correlations of the cap thickness.

Plotting the determined PEC thickness versus applied pressure, one gains 2 linear zones as shown in Fig. 4d). The first linear zone is defined by a steep decrease in PEC thickness (−210 nm per kPa (Pascal)) upon pressure increase. In the present case the PDMS has an elastic modulus of ~5 MPa^41^,^42^ and a total outer rim PDMS area of 0.000252 m^2^. The second zone is displaying less decrease in PEC cap thickness of −135 nm per kPa. The reason for the emergence of two zones is that the overall force used in zone one is not very high and the PEC shear force (which is in the kPa range^3^) still plays a role. However in zone two the PEC compression force (which is orders of magnitude below the one of PDMS^3^) is not significant anymore. Alternative effects which could lead to the two compression zones model, such as mechanical trapping of PEC during printing (and therefore distribution of pressure over larger areas), or differences in PDMS surface and bulk elasticity, are regarded minor.

One interesting effect, which is definitely worth mentioning, is that the PEC shrinks ~50% upon drying^43^. Therefore the results in Fig. 4(d) need to be increased by 50% to show the real conditions upon productions. Increasing the thickness values in Fig. 4 by 50% also causes the real values to overlap with the theoretical compression utilizing Hooks law and PDMS elastic modulus of 5 MPa (64.5 μm (real compression times 1.5) versus 63 μm (theoretical compression)).

#### PEC Stability in Aqueous Environments

**Figure 5:**
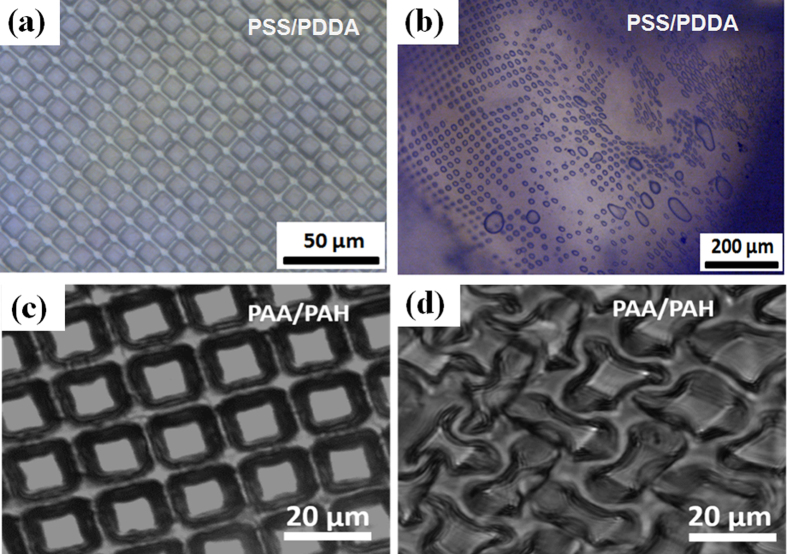
PEC microchambers water stability test using PE concentrations 1:1: LCSM images of PEC. (**a**) PSS/PDDA before and (**b**) after exposure of water (2 mins); (**c**,**d**) (PAA/PAH) microchambers (cross-linked at 150 °C) before (**a**,**d**) after of adding 20 μL of water (stable state after 5 mins).

Due to the deviating molecular structures between PEM and PEC^3^,^44^, the chambers from PEC show a higher sensitivity to aqueous environments (18 °C) compared to those of PEM. This makes the use of PEC in stimuli responsive devices in the future an interesting choice. The produced PEC (PSS/PDDA) or (PAA/PAH) micropatterns are very sensitive towards water, way more than PEM (PSS/PDDA)_60_, see Supplementary Information Fig. S5 and Fig. 5 for comparison. By immersing PEC (PSS/PDDA) into water for several minutes, a complete loss of PEC microchambers patterned structure is observed. On the contrary one needs to carefully measure PEM microchambers thickness chances to see any changes. It is noted that different kinds of PEC as well as crosslinking display completely different stability in water which will be a matter for future publications.

**Figure 6:**
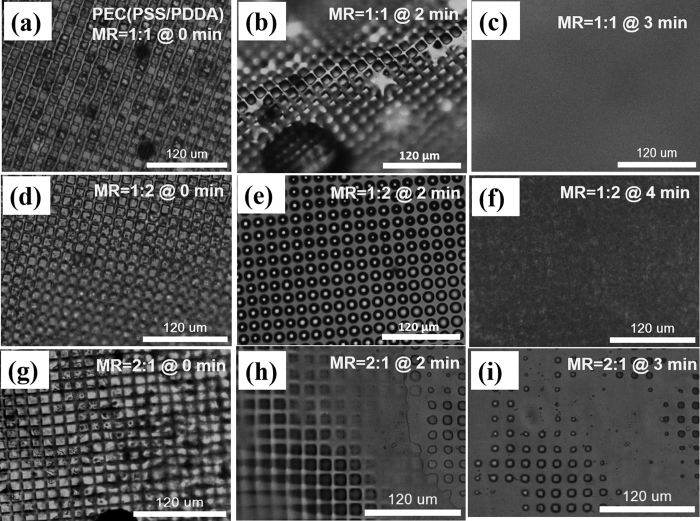
Influence of molar feed ratio (MR) between PSS and PDDA on chamber stability in aqueous environments. The PEC microchambers of MR 1:1 (PSS to PDDA) (**a**–**c**) are within the first 2 mins more stable than in the ratio of 2:1 (PSS to PDDA) (**g**–**i**). Upon 3 mins both chamber types loose structure, whereby air bubbles show in case of PSS excess where the chambers were. An excess of PDDA (1:2) (PSS to PDDA) (**d**–**f**) results into increased PEC stability in water due to PDDA being more hydrophobic than PSS. Microphase separation causes increased scattering and loss of structure in contrast to balanced molar ratio or PSS excess.

Comparing the stability of different molar feed ratios of PECs in water one expects a maximum stability of PECs at feed ratios around 1:1 due to charge balance. In case of PSS-PDDA PECs the maximum stability was determined with excess of PDDA, as shown in Fig. 6. This is due to the hydration water binding to PSS^45^,^46^ and PDDA excess shielding the PSS. Also the PDDA excess seems to introduce in addition to hydration-based structure loss microphase separation due to increasing sample scattering after 3 minutes. This microphase separation is additionally a hint for PDDA acting as a hydrophobization agent for PECs. A charge balance or PSS excess on the contrary leads to increased diffusion and therefore faster loss of structure compared to PDDA excess.

#### Influence of mechanical properties of used PECs

**Figure 7:**
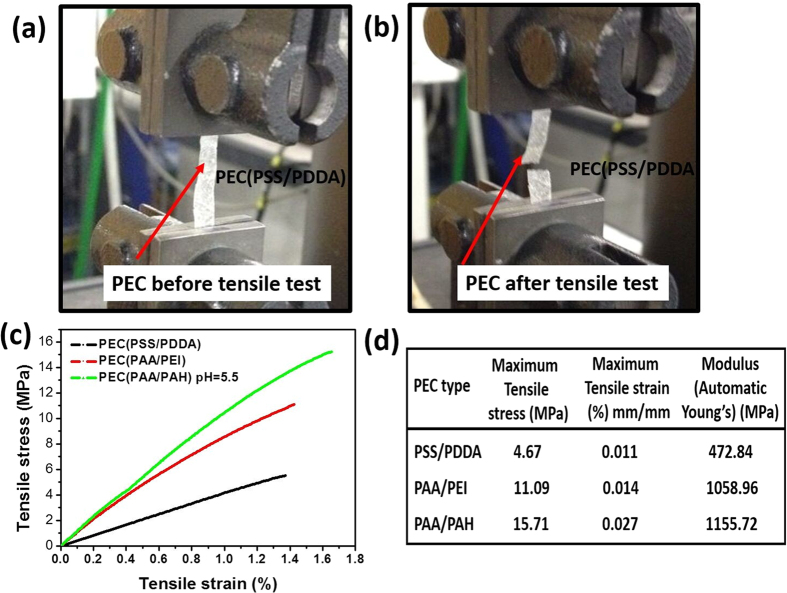
PE concentration 1:1 measurements (**a**) PEC (PSS-PDDA) film in the tensile testing device, (**b**) PEC film ripped after the test, (**c**) Force-deformation curves for PECs made of PSS-PDDA, PAA-PEI and PAA-PAH, (**d**) maximum tensile stress, modulus and strain of PSS-PDDA, PAA-PEI and PAA-PAH PEC.

PEC made from PSS-PDDA, PAA-PAH and PAA-PEI show completely different mechanical properties, as Fig. 7 shows. Despite the maximum elongation and mechanical strength deviating by factor 2.5, the casting quality is comparable. Figure 7 shows the 3 different PECs which display completely different mechanical properties whereby PSS-PDDA PEC was ~3 times softer than PAA-PAH PEC. An interesting phenomenon is that PAA-PAH based PEC is ~2.5 times more elastic (see corresponding maximum elongation in Fig. 7(d). This finding is especially surprising, since usually harder materials are more brittle, which is also in line with PEM and PEM composite films^47^.

The authors speculate that rebinding hydrogen bonds are responsible for the increased hardness as well as elasticity of PAA-PAH PEC compared to PSS-PDDA PEC. In addition the PSS and PDDA have more bulky side groups compared to PAA and PAH, which can explain partly the increased brittleness of PSS/PDDA PEC. For extruded PSS-PDDA PECs elasticity increases but stiffness decreases upon increase of salt concentration^48^. For this reason we expect that for PECs made at a higher ionic strength than used here, thicker PEC caps are needed to prevent chamber collapse^48^. Changing the molar feed ratio of PSS-PDDA from 2:1 over 1:1 to 1:2 significantly changes the mechanical properties of PEC. The maximum toughness and elongation was achieved for balanced molar feed ratios. Increasing excess of either PE decreases maximum elongation as well as maximum mechanical strength as Supplementary Information Fig. S6 shows.

All types of PECs used in this work resulted into the same PEC microstructures when pressing the mould at 60–70 °C on the PECs. This finding is true despite the glass transition temperature for PSS/PDDA PEM (2D PEC) being around 34 °C^33^ while the one for PAA-PAH PEM films is ~120 °C^49^. The reason is probably a different molecular arrangement between PEM and PEC, which is in the case of PEC much more random, less dense and less arranged^3^,^35^,^38^.

## Discussion

We reported a method to fabricate free-standing hollow PEC-based microchambers by a one-step casting with small applied pressure. The presented approach is significantly faster and allows production of larger defect-free patterns compared to layer-by-layer self-assembly and microcontact printing based microchamber production. Furthermore, we demonstrated that the “cap” thickness of PEC microchambers depends on the applied pressure and can be adjusted. On the contrary both the cap and wall thickness of patterned PEM microchambers are controlled with the numbers of PEM bilayers, as already described elsewhere^9^,^10^.

The presented method for PEC one-step casting with applied pressure is due to the (largely) surface force independent production process especially useful to prepare polyelectrolyte-based structures, which could not be produced until now. In addition, this method is significantly faster, especially since a single mould may be re-used multiple times. This allows one to produce a large number of PECs, which can be cast when needed, speeding up the PEC microstructures production. Such a “one-go” approach is not possible for PEMs. It is worthy of note that this was proven for all tested PEMs and PECs (all structures could be produced with similar structure resolution for all tested PEC combinations).

PEM thin films show completely different effects and behaviors compared to PEC. For example metal nanoparticles which introduce conductivity, as well as light-responsive properties have to be incorporated as stratified layers into PEM^50^. PE within linearly growing PEM only diffuse vertically up to 3 bilayers, causing limited interdigitation^44^,^51^. One effect until now not investigated for PEC is, whether PEs within it are distributed in all dimensions equally, or if they exhibit a surface structuring comparable to PEMs which offer a 2-zone behavior upon deposition^52^,^53^,^54^.

PEM microchambers are made of a thin shell (shell is much thinner than the dimensions of microchambers, with a free space between isolated microchambers) while PEC microchambers are embedded into a PEC membrane (the space between microchambers is filled with PEC). This makes PEC structures much more robust minimizing risks of reservoir collapse followed by burst release of the entire drug payload, the event that could be catastrophic, e.g. if occurring not at a defined time on an implant. At the same time, thin shells of isolated PEM chambers have much higher surface area compared to a PEC cap of similar thickness. This should make diffusion of small molecules/ions much faster through PEM than PEC microchambers, thus reducing the responding time for sensor applications. Due to the present advantages the authors will pursue the investigation of PEC properties further.

## Methods and Materials

### Materials

Poly(allylamine hydrochloride) (PAH, M_W_ 58,000), poly(4-styrenesulfonate) sodium salt (PSS, M_W_ = 70,000), poly(acrylic acid) (PAA, M_W_ 100,000), poly(diallyldimethylammonium chloride) (PDDA, M_W_ 200,000–350,000), poly(ethylenimine) (PEI, M_W_ ~750,000) were all purchased from Sigma (St. Louis, USA). Poly(dimethylsiloxane) (PDMS) kit (Sylgard 184) was purchased from Dow-Corning, Midland, USA. In addition PDMS (Elastosil, RT 602) from Wacker AG, München, Germany was used. Deionized (DI) water from a Milli-Q (Millipore) water purification system was used to make all solutions. All the chemicals were used as received without further purification.

### Patterned PDMS moulds

PDMS moulds 1 were prepared by casting PDMS pre-polymer and curing agent (10:1 ratio) onto silicon masters (prepared by traditional photolithography and etching processes), degassing it for 30 minutes in vacuum and curing it at 70 °C (Sylgard 184 needed 3 hours for curing, Elastosil RT 602 needed 1 hour). PDMS moulds 2 were produced by etching PDMS moulds 1 for 15 minutes in oxygen plasma (which creates a layer of silica on the PDMS surface)^55^,^56^ followed by immersing PDMS moulds 1 in PDMS pre-polymer and curing it in similar conditions as used for PDMS moulds 1 fabrication. In the following step PDMS moulds 2 can be lifted from PDMS moulds 1. The PDMS mould creation is illustrated in Supplementary Information Fig. S1(a). Silicon masters with three types of surface features were used in this work: (1) blunted spherical cones 4 μm tall, with larger and smaller base diameters of 10 and 7 μm, respectively, and 20 μm pitch size; (2) an array of blunted cones 10 μm tall, with 5 μm diameter of the larger base and smaller top base shaped as a square having 3 μm edges, the top pitch size is 2 μm; (3) 10 μm tall truncated square pyramids, having larger and smaller base edges of 14 and 10 μm, respectively, and 2.5 μm top pitch size. Examples of PDMS moulds from these master structures can be observed in Supplementary Information Fig. S1.

### PEC complexes

All PE solutions were prepared at a concentration of 2 g/L using an ionic strength of 2 M NaCl (for PAA-PAH and PSS-PDDA PEC of lower ionic strengths down to 0.25 M NaCl were used successfully as well). Two oppositely charged polyelectrolyte solutions (100 mL each) were added at the same time into an empty beaker which was stirred at 500 RPM using a magnet stirrer (Ika, C-MAG-HS10, Staufen, Germany). After around 10–15 minutes of stirring at room temperature (500 RPM), the PECs were harvested. This was achieved by either collecting the agglomerated PEC with tweezers, or letting the PEC precipitate sediment, decant the supernatant water and knead the PEC together. The molar feed ratios in the PE solution mixtures were for PAA-PAH 1:077; PSS-PDDA 1:1.27; PEI-PAA 1:0.6. For mechanical as well as stability comparison measurements molar feed ratios of 2:1, 1:1 and 1:2 for PSS-PDDA PEC were used. In case of PEI-PAA PEC, the PEC formed large fraction of foam, whereby the submerged PEC was extremely sticky and had to be mechanically removed from beaker and magnet stirrer.

After PEC formation 0.2 ± 0.05 g was distributed in a wet state onto PDMS moulds and heated on a heating plate to ~70 °C. After drying ~0.08 g micro-structured PEC is left, proving water content of ~60% in wet PEC. In addition a flat PDMS sheet was placed on top and weights were placed on the flat PDMS sheet to facilitate even pressure distribution and at the same time low adhesion. The PDMS sheet itself (weight ~5 g) introduced a pressure of several Pa on the sample. For stability tests also heat crosslinked PECs were produced, whereby PAH-PAA PECs were simply heated to 150 °C to expel water and create amide crosslinks as explained in detail in ref. 57.

### PEM production

Polyelectrolyte multilayers were prepared by a standard Layer-by-Layer self-assembly method^9^,^58^ using a dip-coating robot similar to ref. 59. PDMS mould 1 was submerged into PEI solution (for all PE solutions the concentration of PE was 2 g/L PE and the ionic strength 2 M (NaCl), same concentrations were used for PEC fabrication) for 10 minutes as the anchoring layer^60^; further alternating layers of PSS and PDDA (alternatively PSS and PAH) were introduced followed by three washings with DI water to remove all non-adsorbed macromolecules. 10 min and 30 s for each adsorption and washing steps were set in program of the dip-coating robot.

The microcontact printing was performed by applying pressure (placing weights on top of sample) (10 kPa) for 1 hour onto the PEM coated PDMS which transfers the PEM to a 20 bilayer PEM (PSS/PDDA)-coated silicon substrate at 100% relative humidity and at room temperature. The PEM bilayers on the glass and silicon substrates were assembled to increase the contact area between patterned PEM and substrate and to improve adhesion to ensure successful transfer^17^,^28^. The PEM chamber production process is shown in Supplementary Information Fig. S1(b).

### Characterization

For electron microscopic investigations, a FEI Quanta ESEM, electron microscope from FEI, Hilsboro, USA was used. Prior to imaging, a 10 nm (~60 seconds sputtering time) film of gold was sputtered onto the samples. Fourier transformed infrared spectroscopic (FTIR) (medium IR range) measurements were performed using a Bruker, Tensor 27, (Bruker, Billerica, MA, USA). Mechanical tests of 0.5 cm wide and 4 cm long and 2 mm thick PEC samples were performed using a Instron 5566 (Instron, Northwood, MA, USA), dual column tensile meter, the used elongation speed was 1 mm/S.

## Additional Information

**How to cite this article:** Gai, M. *et al*. Patterned Microstructure Fabrication: Polyelectrolyte Complexes vs Polyelectrolyte Multilayers. *Sci. Rep.* **6**, 37000; doi: 10.1038/srep37000 (2016).

**Publisher’s note:** Springer Nature remains neutral with regard to jurisdictional claims in published maps and institutional affiliations.

## Supplementary Material

Supplementary Information
