# Supplementary material for: Patterned Microstructure Fabrication: Polyelectrolyte Complexes vs Polyelectrolyte Multilayers
Source: Sci Rep. 2016 Nov 10;6:37000. doi: 10.1038/srep37000 (PMC5103270; doi:10.1038/srep37000)
Supplement: Supplementary Information [file srep37000-s1.pdf]

Supplementary Material of: Patterned Microstructure Fabrication: Polyelectrolyte Complexes vs Polyelectrolyte Multilayers

Meiyu Gai[1,2], Johannes Frueh[1*], Valeriya L. Kudryavtseva[3], Rui Mao[2], Maxim V. Kiryukhin[3], Gleb B. Sukhorukov[2*]

[1]Micro/Nano Technology Research Centre, Harbin Institute of Technology, Yikuang Street 2, Harbin 150080, China

[2]School of Engineering and Materials Science,Queen Mary University of London, Mile End, Eng, 215, London E1 4NS, United Kingdom

[3] National Research Tomsk Polytechnic University, Department of Experimental Physics, Tomsk 634050, Russia

[4]Institute of Materials Research and Engineering (IMRE), A*STAR, 2 Fusion polis Way, Innovis, 08-03, 138634, Singapore

Supplementary Figures

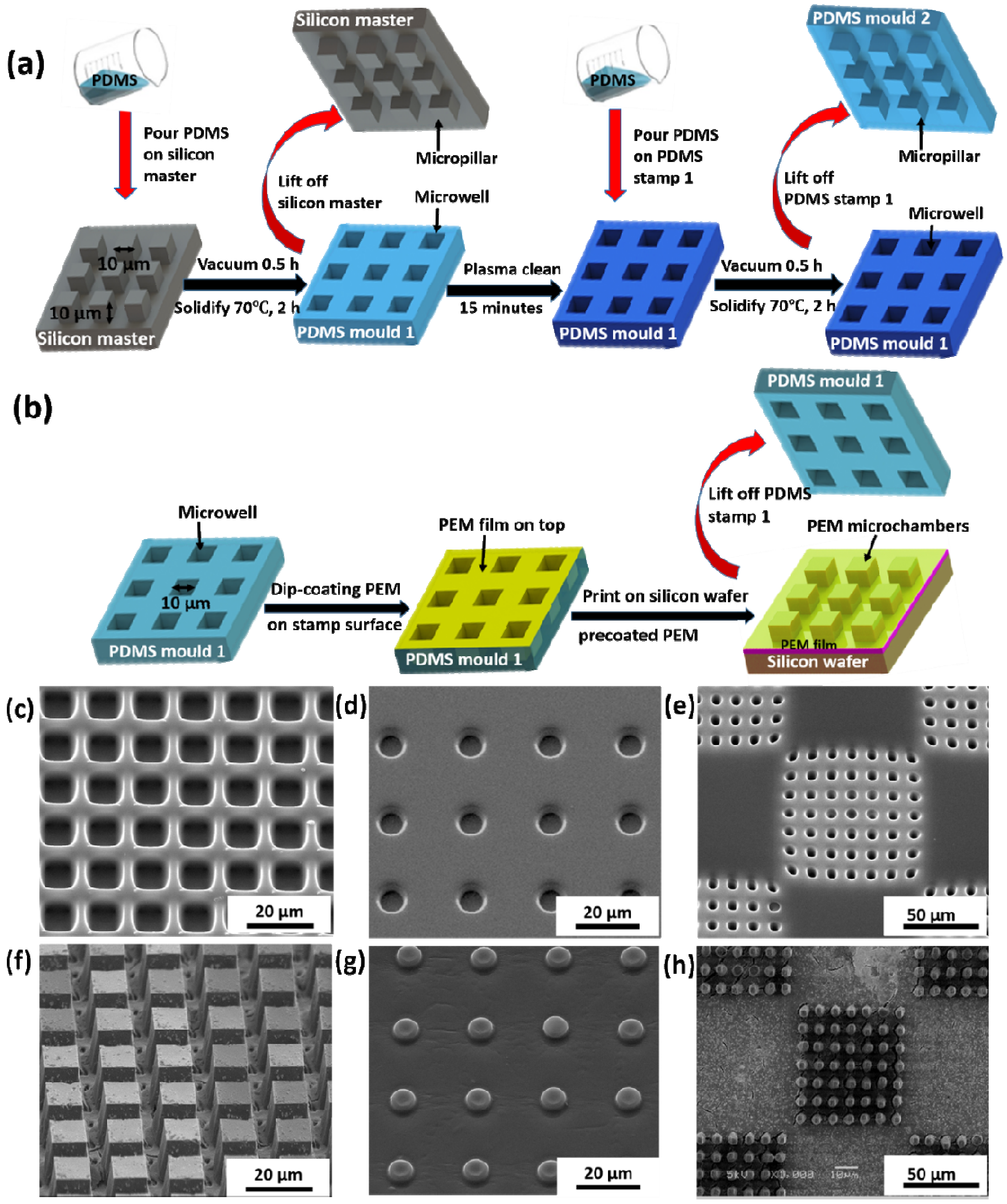

Figure S1. a) Scheme of PDMS mould 1 and PDMS mould 2 creation via PDMS casting and plasma etching based on silicon master; b) Scheme illustrating the fabrication of polyelectrolyte multilayers (PEM) microchambers; (c, d, e) Scanning

electron microscope (SEM) images of PDMS mould 1 made from silicon master with square, round or truncated pyramidal shape microwell patterns, (f, g, h) SEM images of corresponding patterned PEM $(PAH/PSS)_{60}$ 3D hollow microchambers on substrate (pre-coated with PEM $(PSS/PDDA)_{20}$) after transferring the PEM microchambers from PDMS mould 1.

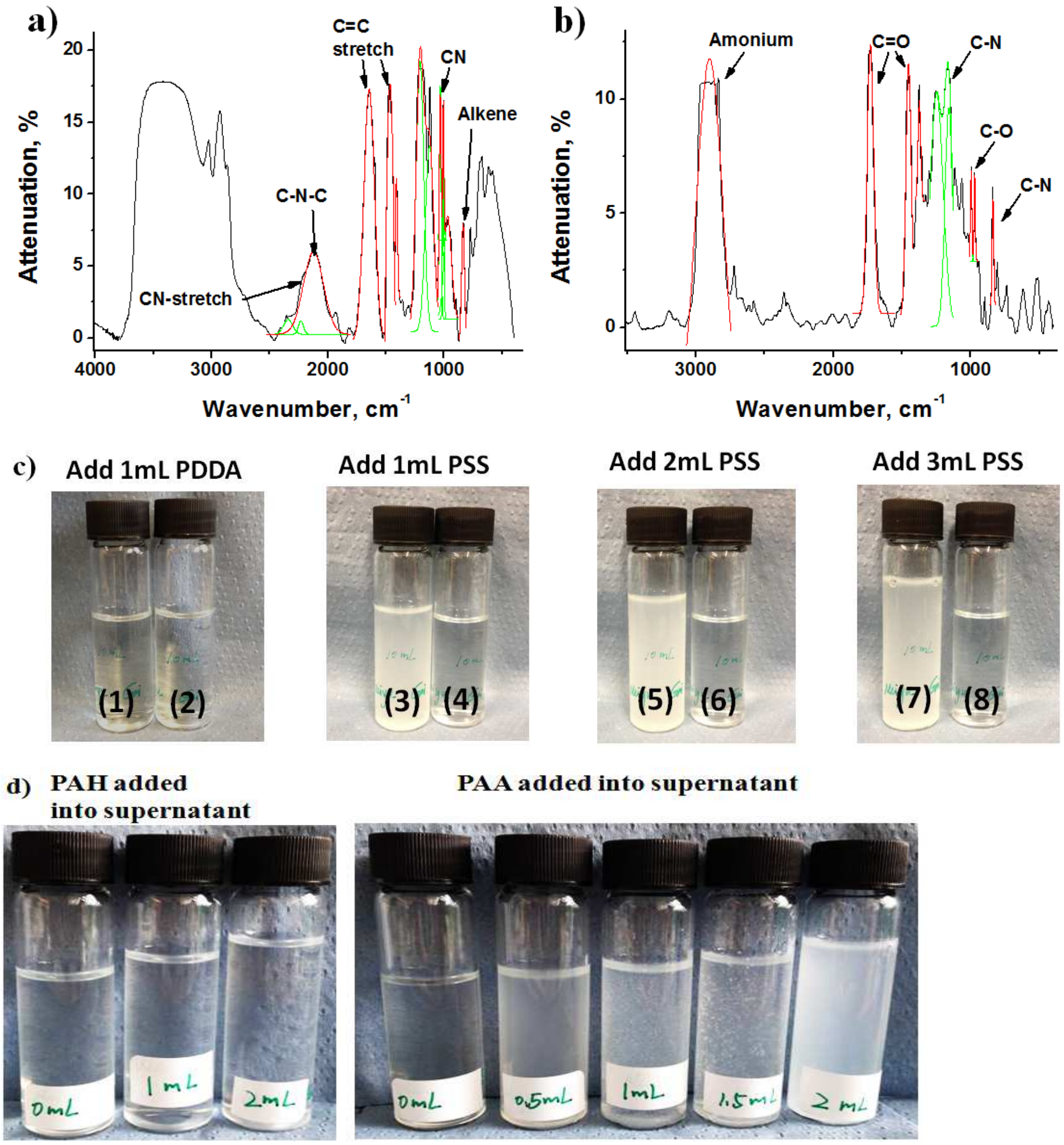

Figure S2. FTIR spectra of a) PSS-PDDA PEC and b) PAA-PAH PEC. The identified, fitted and used peaks are labeled. Peak areas were used to compare the ratio between polyanion and polycation. c) displays the effect of adding PDDA and PSS solution to supernatant solution of produced PECs. Adding PDDA shows no effect, while adding PSS causes PEC aggregation. Adding PSS in access causes dissolution of PEC aggregates, due to charge overcompensation, (1, 4, 6, 8) are solutions after centrifugation and (2) is PDDA 1ml added into supernatant, (3, 5, 7) are solutions after adding PSS 1ml, 2ml, 3ml into supernatant. d) shows the effect of adding either PAH or PAA into the supernatant of the PEC solution, whereby adding access of PAA again causes dissolution of PEC.

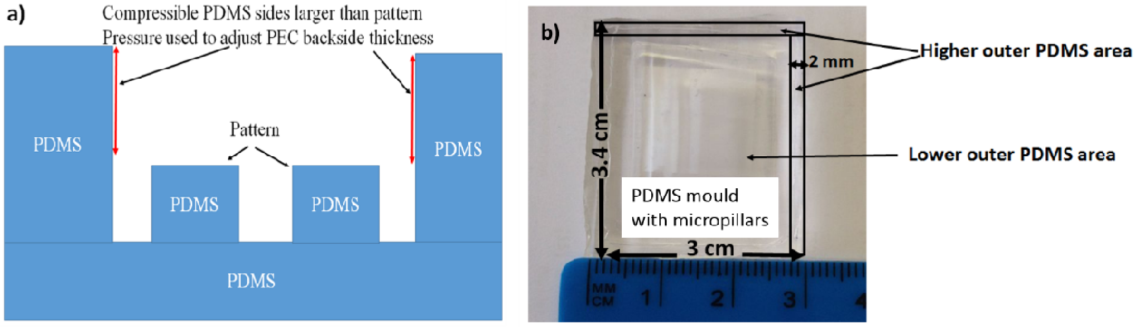

Figure S3. Adjustment of PEC backside thickness: Due to PDMS being linear compressible the thickness of PEC backside can be adjusted by pressure and compressing features exceeding the PDMS pattern. These additional patterns were intentionally left over (size and area known) from PDMS production and defined by silicon mould thickness. PEC thickness between patterns can be adjusted from 1mm thickness until grating (no PEC between patterns). a) Scheme of PDMS mould for thickness adjustment, b) photograph of real stamp.

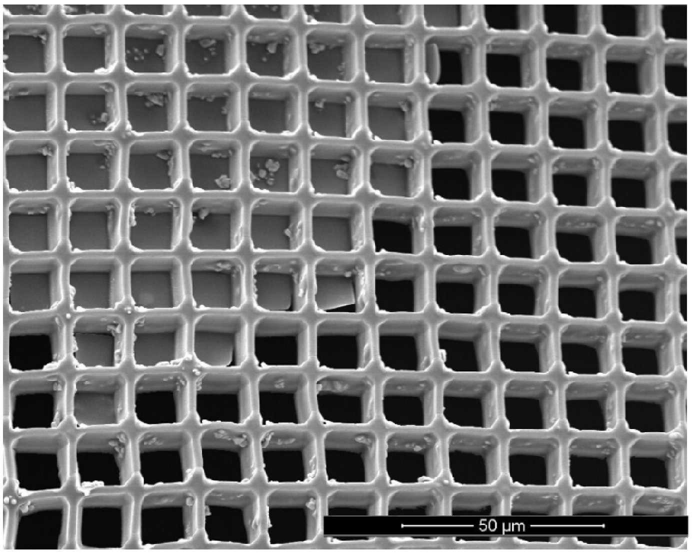

Figure S4. Grating like PEC from square pattern. Due to linear and not isotropic pressure distribution caps of linear increasing thickness can be produced as well.

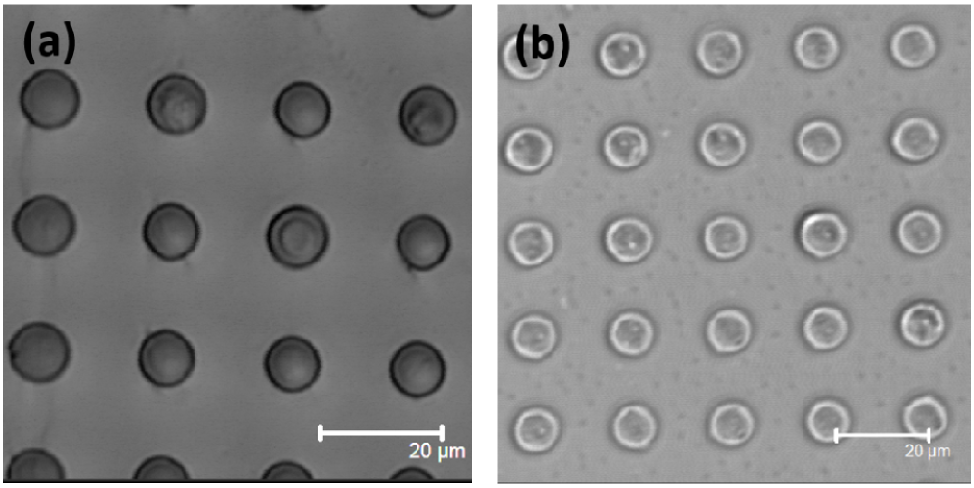

Figure S5. Laser Confocal Scanning Microscope (LCSM) image of PEM PEI(PSS/PDDA)$_{60}$ microchambers on glass slide pre-coated with PEM PEI(PSS/PDDA)$_{20}$, before adding water in dry condition a), after adding water after 30mins b), the hollow microchambers still keep the shape.

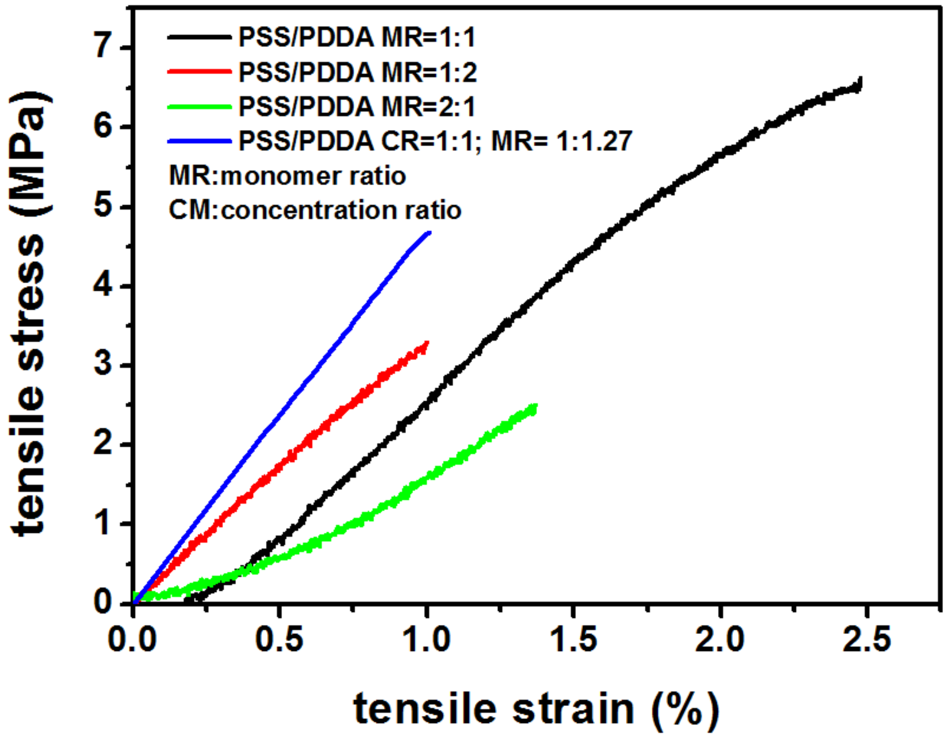

Figure S6. Influence of molar feed ratio of monomer groups of PSS and PDDA on tensile strain and tensile stress. A balanced ratio achieves maximum tensile strain and stress while imbalance of the molar feed ratio decreases the maximum tensile strain and stress.
